# Synthesis of cyclic α-pinane carbonate – a potential monomer for bio-based polymers†

**DOI:** 10.1039/d1ra07943c

**Published:** 2022-06-13

**Authors:** Valentine C. Eze, Abdul Rehman, Manthan Patel, Sajjad Ahmad, Adam P. Harvey

**Affiliations:** School of Engineering, Newcastle University Newcastle upon Tyne NE1 7RU UK; Department of Chemical and Polymer Engineering, University of Engineering and Technology Lahore Faisalabad Campus Pakistan a.rehman2@uet.edu.pk

## Abstract

This work reports the first known synthesis of α-pinane carbonate from an α-pinene derivative. Pinane carbonate is potentially useful as a monomer for poly(pinane carbonate), which would be a sustainable bio-based polymer. α-Pinene is a major waste product from the pulp and paper industries and the most naturally abundant monoterpene in turpentine oil. α-Pinene is routinely converted to pinene oxide and pinanediol, but no study has yet demonstrated the conversion of pinanediol into α-pinane carbonate. Here, α-pinane carbonate was synthesised *via* carboxylation of α-pinanediol with dimethyl carbonate under base catalysis using triazabicyclodecene guanidine (TBD). 81.1 ± 2.8% α-pinane carbonate yield was achieved at 98.7% purity. The produced α-pinane carbonate was a white crystalline solid with a melting point of 86 °C. It was characterised using FTIR, NMR, GCMS and a quadrupole time-of-flight (QTOF) mass spectrometer. The FTIR exhibited a C=O peak at 1794 cm^−1^ confirming the presence of a cyclic carbonate. GCMS showed that the α-pinane carbonate fragments with loss of CO_2_, forming pinene epoxide. Base hydrolysis of the α-pinane carbonate using NaOH/ethanol/water regenerated the pinanediol with formations of Na_2_CO_3_.

## Abstract 2

Synthesis of α-pinane carbonate from an α-pinene derivative.

## Graphical abstract

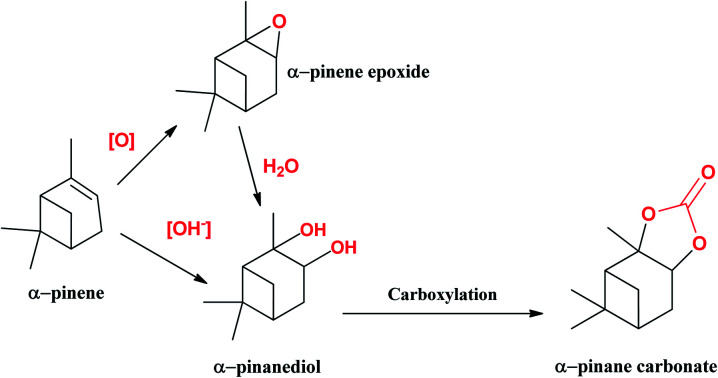

## 1. Introduction

**Scheme 1:**
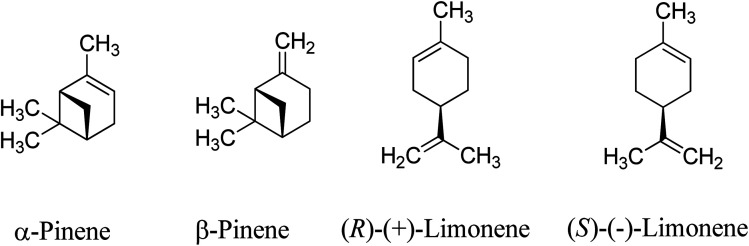
Common monoterpenes found in turpentine oil.

Monoterpenes are naturally occurring unsaturated hydrocarbons obtained as waste products from the pinewood pulping industry. The most commonly available monoterpenes are pinene and limonene, which are cyclic monoterpene isomers comprising two iso-propene units with a molecular formula of C_10_H_16_ (Scheme 1). Approximately 3.5 × 10^5^ tons per year of turpentine oil is produced from the pulping industry globally,^1,2^ with the global supply of turpentine oil expected to increase as the pulping industry grows. The pulping process produces between 0.3 and 1.0 kg of turpentine oil per tonne of pulp,^3^ of which the predominant monoterpene is α-pinene (∼70%) when using the current sulphate processes.^3,4^ Limonene accounts for ∼8.6% (0.3 × 10^5^ tons per annum) of the worldwide turpentine oil production.^1^

**Scheme 2:**
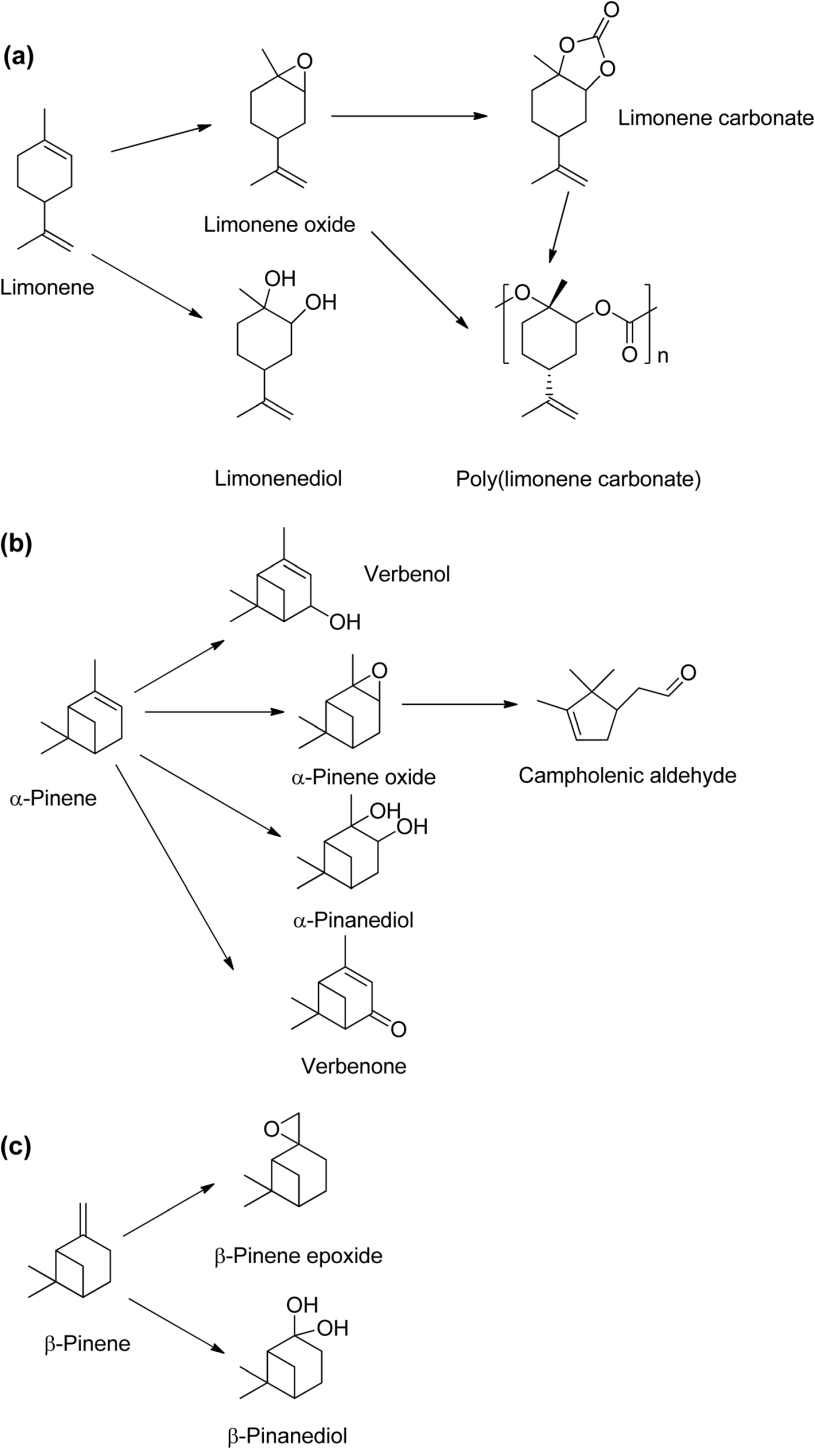
Chemical compounds derived from monoterpenes found in turpentine oil, (a) limonene, (b) α-pinene and (c) β-pinene.

Crude turpentine oil from steam distillation of pine is typically 75–85% α-pinene, and the remainder were 0–3% β-pinene and 5–15% limonene.^5^ A major source of limonene is the peel of citrus fruits which contains about 90 wt% limonene.^6^ Limonene is mainly obtained from the waste products of orange harvesting and peel from orange juice production.^7^ The large global tonnage of α-pinene and limonene could provide substantial amounts of sustainable platform chemicals for bio-based polymer productions.^8^ There have been extensive studies of limonene derivatives such as limonene epoxide^9–11^ and limonene bis-epoxides,^12,13^ which can be copolymerised with CO_2_ to obtain poly(limonene carbonates),^9,11,14–16^ as shown in Scheme 2(a). Limonene oxides and carbonates can be used as highly valuable platform chemicals for biopolymer synthesis.^17–20^ Conversely, despite α-pinene being the most abundant naturally occurring monoterpene,^17^ there are limited studies on transformations of α-pinene into essential derivatives, such as α-pinene epoxide and α-pinane carbonate.

The α-pinane carbonate is potentially a monomer *via* a ring-opening copolymerisation to produce poly(α-pinane carbonate). The α-pinene epoxide is an important intermediate for other chemicals including campholenic aldehyde used in fragrances^8,21,22^ and pinanediol.^23^ α-Pinene can also be oxidised to produce essential chemicals such as verbenol and verbenone (Scheme 2(b)).^8,24,25^ Unlike limonene epoxide, which can be readily converted to limonene carbonate, for potential industrial productions of poly(limonene carbonate), the applications of α-pinene oxide have been mainly limited to its isomerisation products such as campholenic aldehyde,^22^ as no route from α-pinene oxide to the carbonate has yet been established.

Various attempts to synthesise polyether *via* homo-polymerisation of α-pinene oxide or poly(α-pinene carbonate) from α-pinene epoxide and CO_2_ have been unsuccessful. No poly(α-pinane carbonate) or α-pinane carbonate was obtained in the reactions of α-pinene epoxide with CO_2_.^26^ A recent review on the copolymerisation of sustainable epoxides with CO_2_ also reported that there are currently no available publications on the successful copolymerisation of α-pinene oxide with CO_2_.^27^ However, an existing patent claims that poly(pinene carbonate) can be produced by the reaction of pinene epoxide and CO_2_ in the presence of a (salen)CrCl/PPNCl catalyst.^27^ The lack of reactivity of the α-pinene epoxide in ring-opening polymerisation has been attributed to a higher reaction barrier of the α-pinene epoxide in the ring-opening process due to steric hindrance.^26^ The lack of reactivity is unfortunate, as there is potentially a significant market for pinane carbonate, given that the current production of cyclic carbonates is 4 million tons per year.^28^ This could add value to the paper and pulp industries, whilst introducing a new sustainable platform chemical. The cyclic pinane carbonate could be polymerised using established methods for ring-opening polymerisation of cyclic organic carbonates.^29–31^

This study reports the first synthesis of α-pinane carbonate from α-pinanediol. The synthesis route reported here involved a novel and eco-friendly process *via* reactions of α-pinanediol with dimethyl carbonate (DMC). Reactions of diols with DMC to obtain a cyclic carbonate have been reported for some other polyhydric alcohols,^32^ but have not previously been applied for α-pinanediol. The only existing report of pinane carbonate synthesis was *via* the reaction of 2,10-β-pinanediol (Scheme 2(c)) with ethyl chloroformate in pyridine, followed by a molecular rearrangement.^33^ Although 2,10-β-pinanediol could be obtained from hydroxylation of β-pinene, the content of β-pinene in crude turpentine oil is very low, typically about 0–3%, as compared to 75–85% for α-pinene.^5^ The DMC route was chosen for this synthesis because it is less toxic compared to the phosgene process.^34^ It is anticipated that the use of α-pinanediol would reduce the reaction barrier associated with α-pinene epoxide and overcome the steric hindrance,^26^ allowing for formations of α-pinane carbonate. α-Pinanediol can be readily obtained *via* osmium tetraoxide catalysed hydroxylation of naturally available α-pinene,^35^ or from hydrolysis of the α-pinene epoxide.^23^ Furthermore, DMC is a green solvent, which has been synthesised by CeO_2_ – catalysed reactions of CO_2_ and methanol (MeOH) to achieve >95% methanol conversions and >99% selectivity to DMC.^36^

Similarly, some other main methodologies for DMC preparation currently applied in the industry are oxidative carbonylation of MeOH, urea mediated synthesis and transesterification of ethylene carbonate and propylene carbonate.^37^

## 2. Materials and methods

### 2.1 Materials

Materials used in the experiments were pinane-2,3-diol (99%, Sigma-Aldrich), dimethyl carbonate (99%, Sigma-Aldrich), anhydrous methanol (99.8%, Sigma-Aldrich), absolute ethanol (99.5%, Fisher Scientific), sodium hydroxide (97%, Sigma-Aldrich), acetic acid (99%, Sigma-Aldrich), *n*-hexane (95%, Sigma-Aldrich), methyl heptadecanoate (99%, Sigma-Aldrich), and TBD guanidine (98%, Sigma-Aldrich), these chemicals were used as supplied.

### 2.2 Experimental procedures

**Scheme 3:**
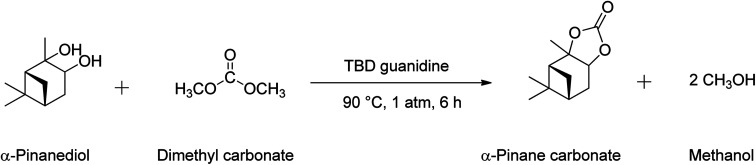
Reaction equation for the synthesis of α-pinane carbonate using 0.18 mol of DMC, 0.03 mol of pinanediol, and 2 mmol of TBD guanidine catalyst.

The pinane carbonate synthesis was performed in a 150 mL 2-neck batch reactor equipped with a condenser and a thermocouple/sampling unit, by reactions of pinanediol and DMC, as shown in Scheme 3. Synthesis of α-pinane carbonate was carried out using 0.18 mol of DMC, 0.03 mol of pinanediol, and 2 mmol of TBD guanidine catalyst. The reaction was carried in the round bottom flask. Pinanediol (5 g) and DMC (15.88 g), corresponding to a 6 : 1 DMC to pinanediol molar ratio, were added to the reactor and heated to the reaction temperature of 90 °C using a heater-stirrer (IKA® Basic). This was followed by additions of 250 mg of TBD guanidine catalyst (5 wt% based on the pinanediol) and mixing at 600 rpm. The reaction mixture was refluxed at 90 °C for 6 h in the reactor. 0.25 mL samples were collected every 1 h and analysed by gas chromatography (GC) to monitor the progress of the reaction until the pinanediol peak disappeared.

The reaction mixture was then neutralized using 110 μL of acetic acid. Excess DMC and residual methanol by-products in the reaction were removed by rotary evaporation at 60 °C at 120 mmHg pressure. An amber-coloured solid was obtained, and this was purified by re-crystallization 3 times in methanol (25 mL). The recrystallized product was dissolved in a mixture of 25 mL *n*-hexane and 10 mL methanol with vigorous shaking. The hexane extract was a colourless viscous solution, from which white crystals of α-pinane carbonate appeared as the excess hexane evaporated at room temperature. The α-pinane carbonate was filtered out and dried at room temperature. The reaction mixture was recrystallized three times to remove any traces of the catalyst. About 4 g of α-pinane carbonate was obtained, with >98.7 wt% purity as confirmed by the GC. The product was characterised using NMR (^1^H and ^13^C), FTIR, GCMS, a differential scanning calorimeter (DSC) and a quadrupole time-of-flight (QTOF) mass spectrometer.

The reactivity of the synthesised α-pinane carbonate was investigated, especially to find a route for regenerating the pinanediol. This is important in ensuring that a suitable process exists for recycling pinanediol from the α-pinane carbonate at the product's end of life. About 0.5 g of the dried α-pinane carbonate was hydrolysed in the batch reactor with 25 mL of 0.5 M NaOH in an ethanol–water solution containing 10 (v/v)% deionised water. The 25 mL 0.5 M NaOH solution was heated in the reactor to 60 °C, followed by the transfer of 0.5 g α-pinane carbonate into the reactor and vigorous mixing at 600 rpm. The ratio of α-pinane carbonate to NaOH solution (w/v) in this study was adapted from the commonly used method for saponification of fats and oils.^38–40^ About 0.5 mL of the reaction sample was collected using a micropipette at various time intervals from 0–60 min. The sample was transferred into a pre-weighed 2 mL vial containing 14 μL of acetic acid to quench the reaction immediately.

### 2.3 Sample analysis

FTIR analysis of the samples was performed using a Mettler Toledo ATR-FTIR spectroscopy (React IR 4000) equipped with a DiComp diamond K6 conduit 16 mm probe. The spectra of the product samples were collected over the range 4000–650 cm^−1^. The instrument was initialized by collecting 256 scan background spectra for air and water vapours before collecting the spectra of the samples. ^1^H-NMR and ^13^C-NMR spectroscopy of the samples were recorded on a Bruker Avance III HD spectrometer at 700 MHz using a nitrogen-cooled cryoprobe prodigy™, with methanol-d (CD_3_OD) as a solvent. Melting points of the pinanediol and the produced α-pinane carbonate were determined using a Reichert platform melting point apparatus. DSC analysis was performed using (TA Instruments, DSC Q20 V24.11 Build 124), with a TA Universal analysis software to do the DSC analysis using 3.1–3.4 mg of samples that were carefully weighed into Tzero aluminium pans and sealed with Tzero aluminium lids. The samples were scanned over the temperature range of 25–400 °C at a heating rate of 5 °C min^−1^ and a 50 mL min^−1^ flow of oxygen-free nitrogen for the sample purge.

A 6890 Hewlett Packard Series GC equipped with a Nukol™ fused silica column of 30 mm length, 0.32 mm ID and 0.25 μm film thickness was used to quantify the α-pinanediol and α-pinane carbonate in the samples. The GC oven temperature was programmed from 120 °C initial temperature held for 5 min and ramped at 15 °C min^−1^ to a final temperature of 210 °C and held for a further 10 min (total time of 21 min). The flame ionisation detector (FID) and injector temperatures were set at 260 and 250 °C, respectively, while a helium carrier gas was used at 10 PSI pressure. Quantifications of the α-pinanediol and α-pinane carbonate were based on a calibration curve using standard reagents where correlation data (*R*^2^ > 0.995) was obtained while methyl heptadecanoate was a GC internal standard. The calibration curve is drawn following concentration ranges from 1.0 to 50 mg kg^−1^ of the reference standard. About 100 mg of each sample was measured into a 2 mL GC vial and mixed with 500 μL of methyl heptadecanoate (10 mg mL^−1^ of ethanol), and 1 μL of the sample mixture was injected into the GC using a 5 μL GC syringe (SGE). The sample is run against this calibration curve to estimate its concentration. The α-pinanediol and α-pinane carbonate were also analysed using a GCMS: 7890B Agilent GC coupled to a 5977B mass selective detector (MSD) in full scan mode from 50–520 amu and 70 eV ionisation energy. The 7890B Agilent GC program was held at 50 °C for 2 min initially and ramped to 310 °C at 5 °C min^−1^ (total time of 75 min). Molecular ions of the analytes were determined with a QTOF mass spectrometer. The chromatogram showed a single peak with a major ion on Rt 11.084 with a maximum area covering the purity content. The exact mass measurement is followed by QTOF with the least error and uncertainty incorporation as the findings correlated with other analysis techniques and no impurity is highlighted.

## 3. Results and discussion

### 3.1 Productions and characterisation of the α-pinane carbonate yield

**Fig. 1:**
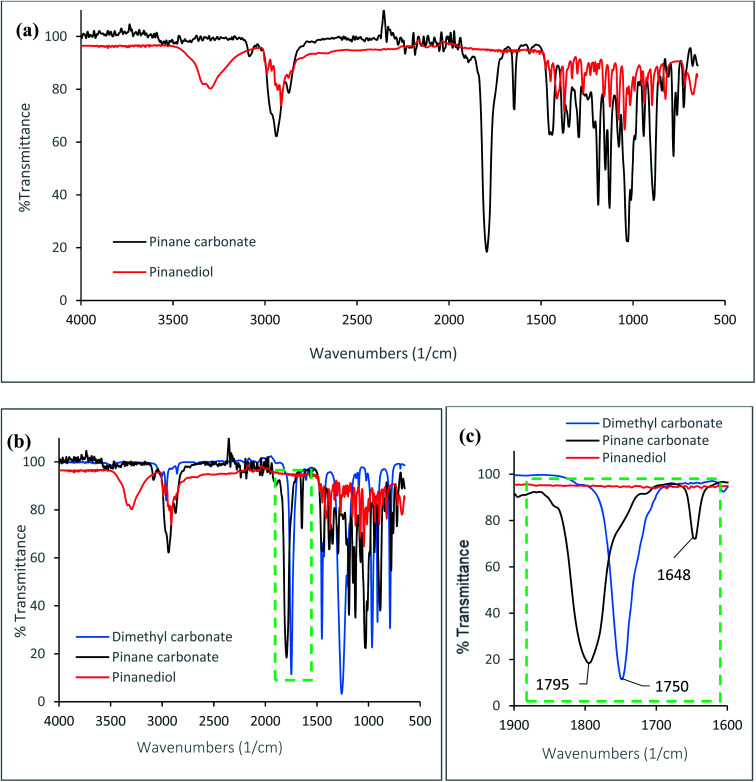
FTIR data collected using a Mettler Toledo React IR 4000 ATR-FTIR spectroscopy equipped with a DiComp diamond probe, (a) for the α-pinanediol and the pinane carbonate, (b) for α-pinanediol and pinane carbonate, with FTIR of DMC inserted as a typical linear carbonate, (c) shift in absorption wavelengths for cyclic and linear organic carbonates.

An α-pinane carbonate was synthesised from an α-pinene derivative for the first time, *via* reactions of α-pinanediol and DMC. The cyclic α-pinane carbonate yield for the process was 81.1 ± 2.8%, and the pinane carbonate was isolated as a white crystalline solid at 98.7% purity as confirmed by the GC. The melting points of the α-pinanediol and the α-pinane carbonate were determined by DSC, using methods reported elsewhere,^41^ and these values were 86 °C for the α-pinane carbonate, and 55 °C for the 2,3-pinanediol (ESI Fig. 1†). FTIR data for the α-pinane carbonate and the α-pinanediol are shown in Fig. 1. Fig. 1(a) shows an FTIR peak around 3200–3300 cm^−1^, which is assigned to the O–H stretching vibration for the α-pinanediol. This peak was conspicuously absent in the α-pinane carbonate product. The disappearance of the O–H peak resulted in the formation of a new peak at 1795 cm^−1^, corresponding to C=O stretching vibration for cyclic carbonates, as shown in Fig. 1(b) and (c), which is significantly higher than the absorption wavenumber of about 1750 cm^−1^ for linear organic carbonate (DMC). The C=O stretching vibration for the α-pinane carbonate compares well with 1800 cm^−1^, which has been reported for limonene cyclic carbonate.^11^ A previous study has also demonstrated that C=O stretching for cyclic propylene carbonate occurs at a higher wavenumber (1799 cm^−1^) than the C=O stretch for linear poly(propylene carbonate) with wavenumber at 1747 cm^−1^.^42^ Apart from the characteristic FTIR peaks at 3200–3300 cm^−1^ for the O–H and 1795 cm^−1^ for the C=O functionalities, other FTIR peaks observed at 2980–2850 cm^−1^ for C–H stretching vibrations in methyl and methylene groups are similar for both species, as would be expected. The FTIR data clearly shows that the α-pinanediol was converted to α-pinane cyclic carbonate during the reaction.

**Fig. 2:**
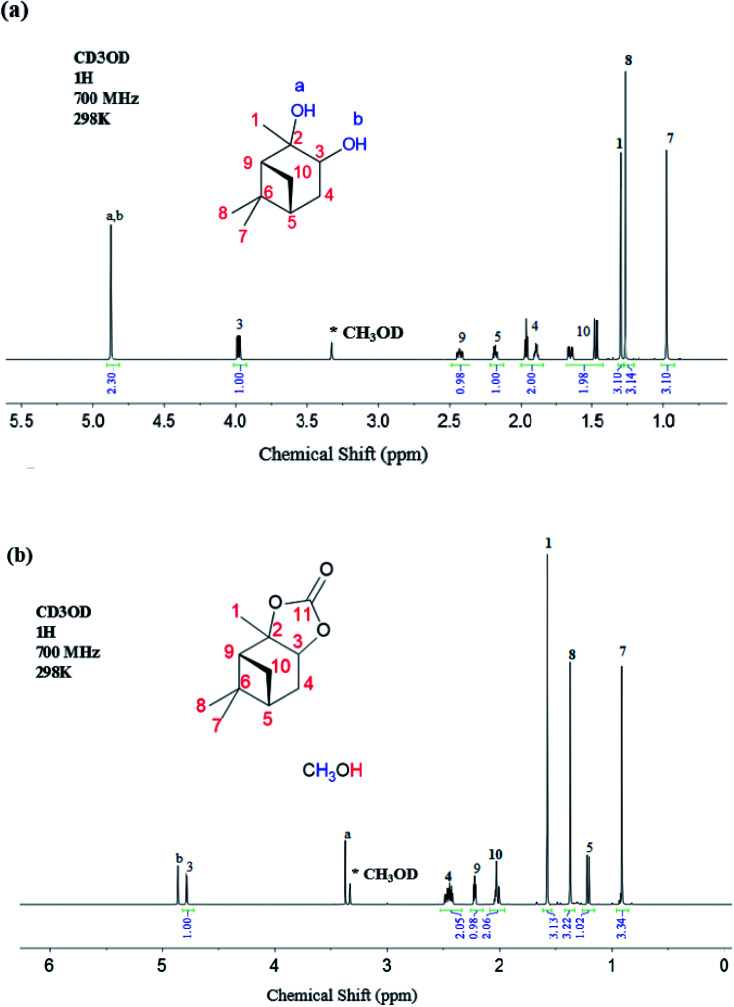
^1^H-NMR spectroscopy recorded on a Bruker Avance III HD spectrometer with nitrogen-cooled cryoprobe prodigy™, with methanol-d (CD_3_OD) as a solvent and operating at 700 MHz (a) α-pinanediol and, (b) α-pinane carbonate.

The ^1^H NMR data for the α-pinanediol and the α-pinane carbonate are shown in Fig. 2. The α-pinanediol has ^1^H NMR peaks (700 MHz, CD_3_OD), as shown in Fig. 2(a), at the following chemical shifts (*δ*/ppm): 1.30 (3H, s), 3.98 (1H, dd), 1.89 (1H,t) for proton that points out of the α-pinane ring (*exo*-proton) and 1.97 (1H, dtd) for the proton that points inside the α-pinane ring (*endo*-proton) which is more shielded, 2.18 (1H, dtd), 0.98 (3H, s), 1.27 (3H, s), 2.43 (1H, dddd), 1H (1.47, m) for *exo*-proton and 1H (2.43, dddd) for the more shielded *endo*-proton, and 4.88 (2H, s) for the –OH groups. The ^1^H NMR peak at 4.88 ppm (2H, s) corresponds to the O–H protons of the pinanediol, which indicates an overlap in the ^1^H NMR signals for the two hydroxyl groups on the pinanediol. A total of 9 hydrogen environments corresponding to a total of 18 hydrogen atoms were observed for the α-pinanediol (C_10_H_18_O_2_).

The formation of α-pinane carbonate resulted in the disappearance of the proton peak at a chemical shift of 4.88 ppm, and slight variations in the chemical shift for some of the proton peaks, as shown in Fig. 2(b). The ^1^H NMR peaks (700 MHz, CH_3_OD) for the α-pinane carbonate were observed at the following chemical shifts (*δ*/ppm): 1.58 (3H, s), 4.78 (1H, d), 2.43 (2H, m), 1.21 (1H, d), 0.91 (3H, s), 1.37 (3H, s), 2.22 (1H, d) and 2.02 (2H, m). ^1^H NMR peaks were also observed at 3.4 ppm (3H, s) and 4.9 ppm (1H, s), due to remaining methanol impurities. Overall, 8 hydrogen environments were observed, corresponding with a total of 16 hydrogen atoms for the C_11_H_16_O_2_ in the α-pinane carbonate. The enantiotropic effect was observed in some of the ^1^H-NMR peaks due to the different chemical environment of protons. For instance, the two protons attached to C4 were in different chemical environments as *endo* and *exo* protons, likewise, the two protons attached to C10. Therefore, the enantiotropic effect accounts for the differences in chemical shifts for the C4 and the C10 protons shown in Fig. 2(a). Similarly, protons on the two methyl groups (C7 and C8) attached to carbon number 6 experience different chemical environments due to the enantiotropic effect. Protons on C7 are more shielded (*δ* = 0.98 ppm), indicating an *endo*-position of the C7 methyl group, while the protons on C8 are more deshielded (*δ* = 1.27 ppm) which suggests that the C8 methyl group occupies an *exo*-position on the α-pinane ring.

**Fig. 3:**
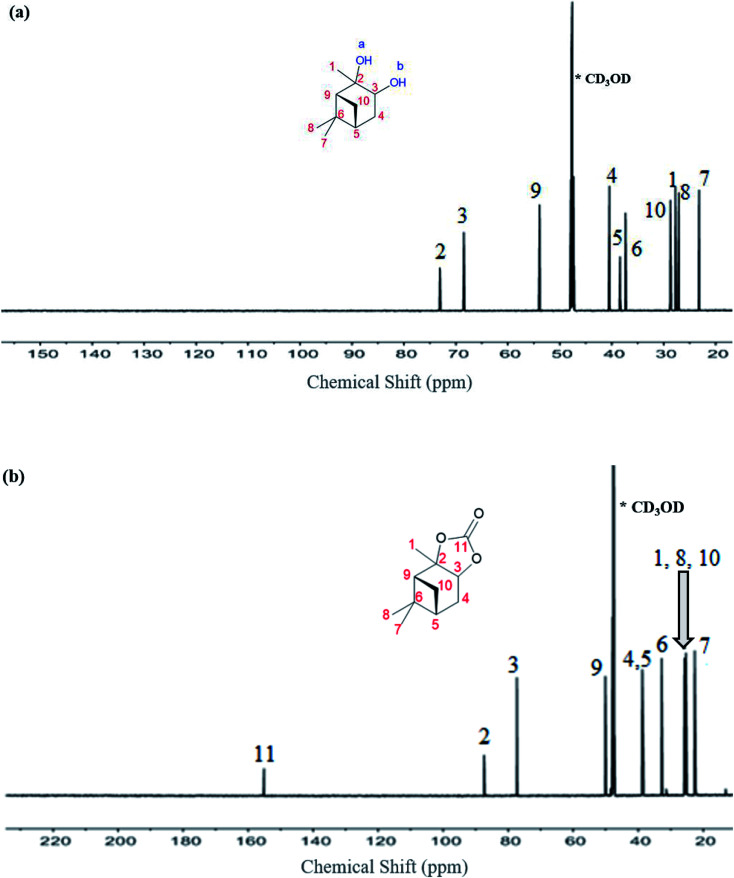
^13^C-NMR spectroscopy recorded on a Bruker Avance III HD spectrometer with nitrogen-cooled cryoprobe prodigy™, with methanol-d (CD_3_OD) as a solvent and operating at 700 MHz, (a) α-pinanediol and, (b) α-pinane carbonate.

The ^13^C NMR data for the α-pinanediol and α-pinane carbonate are shown in Fig. 3. The α-pinanediol has ^13^C NMR peaks (700 MHz, CD_3_OD) at the following chemical shifts (*δ*/ppm): 23.2 (C7), 27.1 (C8), 27.7 (C1), 28.7 (C10), 37.3 (C6), 38.4 (C5), 40.4 (C4), 53.9 (C9), 68.5 (C3), 73.1 (C2). The carbon-13 NMR results in Fig. 3(a) show 10 different carbon environments, corresponding to the various carbon atoms on the C_10_H_18_O_2_ for the α-pinanediol. Mass spectra data (ESI Fig. 2†) from the GCMS analysis of α-pinanediol showed major *m*/*z* peaks at 55.0, 71.0, 99.1 (100%), 111.1, and 126.1, confirming the starting material as (1*S*,2*S*,3*R*,5*S*)-(+)-pinanediol (#7655 in NIST98.1 MS database). No molecular ion peak for pinanediol was observed at 170.3 *m*/*z*, indicating low stability of its molecular ions.

The chemical shifts observed in α-pinane carbonate differed from pinanediol. An additional carbon peak was observed at *δ* (ppm) of 155.1, as shown in Fig. 3(b), corresponding to C=O of the α-pinane carbonate. The carbon peak at 155.1 ppm chemical shift is consistent with C=O carbon for organic carbonates.^9,43^Fig. 3(b) shows that the α-pinane carbonate has ^13^C NMR peaks (700 MHz, CD_3_OD) at the following chemical shifts (*δ*/ppm): 22.6 (C7), 25.3 (C8), 25.3 (C1), 25.7 (C10), 32.8 (C6), 38.4 (C5), 38.7 (C4), 50.1 (C9), 77.3 (C3), 87.4 (C2), and 155.1 (C11). These 11 carbon environments correspond to the various carbon atoms on the C_11_H_16_O_3_ for the α-pinane carbonate.

The major *m*/*z* mass spectra peaks for the α-pinane carbonate (ESI Fig. 3†) were 55.0, 67.0 (100%), 69.0, 83.0, 109.1, and 137.0. A molecular ion peak for pinane carbonate was also not observed at the expected *m*/*z* of about 196.2. Mass spectra for the α-pinane carbonate in ESI Fig. 3† showed that the *m*/*z* peaks are consistent with fragmentation patterns for α-pinene epoxide in the National Institute of Standards and Technology (#21620 in NIST98.1 MS database), indicating that loss of CO_2_ and molecular rearrangement to form α-pinene epoxide was the plausible fragmentation mechanism of α-pinane carbonate. Molecular ions of the α-pinanediol (152.1 g mol^−1^) and the α-pinane carbonate (196.1 g mol^−1^) were subsequently determined as shown in ESI Fig. 4† with the QTOF mass spectrometer.

It is expected that α-pinane carbonate can be polymerised to obtain poly(α-pinane carbonate), which can be used as a bio-based thermoplastic, like poly(limonene carbonates).^9,15^ poly(limonene carbonate) has a substantially high glass-transition temperature (*T*_g_ = 130 °C),^15^ and this is the expected range for a poly(α-pinane carbonate). Therefore, it is envisaged that a poly(α-pinane carbonate) could be a sustainable replacement for petroleum-derived polycarbonates.

### 3.2 Recovery of α-pinanediol *via* base hydrolysis of the pinane carbonate

**Fig. 4:**
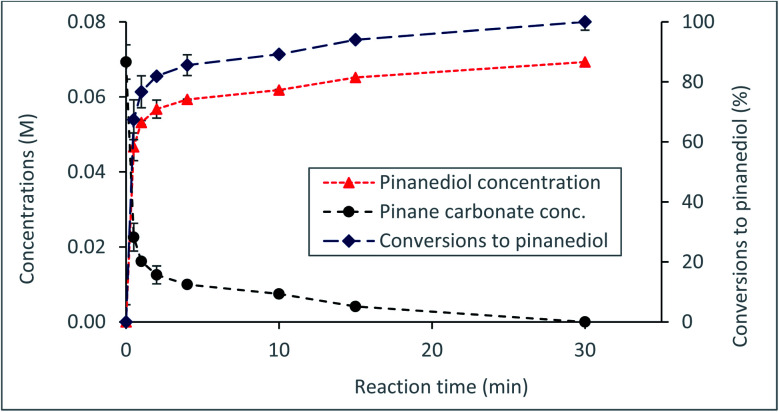
Recovery of α-pinanediol *via* base hydrolysis of the α-pinane carbonate in a batch reactor at 60 °C using 0.5 M NaOH ethanol/water solution (90 : 10 v/v)%.

Currently, a major challenge for the plastics industry is the development of efficient strategies for the handling of waste plastic at the end of life.^44,45^ Proposed routes for waste plastics handling include recycling^44^ or composting, in the case of biodegradable materials, such as bioplastics used in packaging.^45^ However, a more acceptable route would be through recovery and recycling of the starting materials for further utilisation. To develop a feasible route for possible recovery and recycling of the α-pinanediol starting material, the reactivity of the α-pinane carbonate towards base hydrolysis was investigated. The results in Fig. 4 show that quantitative recovery of α-pinanediol was achieved from the base hydrolysis of the α-pinane carbonate. A poly(α-pinane carbonate) is expected to undergo similar reactions.

**Scheme 4:**
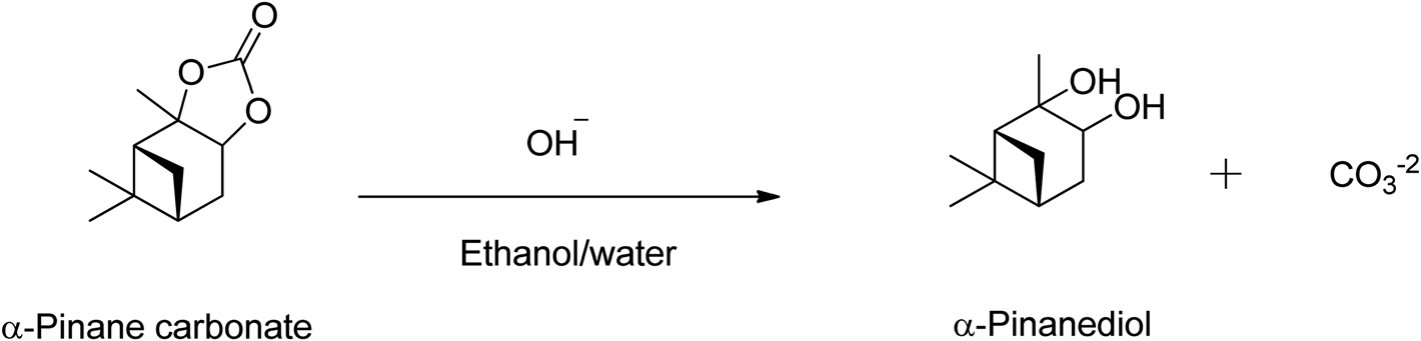
Base hydrolysis of α-pinane carbonate using NaOH in ethanol/water.

Over 80% of the pinane carbonate was saponified within 2 min using 0.5 M NaOH prepared in ethanol/water solution containing 90 : 10 (v/v)% and 60 °C. At about 30 min reaction time, all the pinane carbonate had been completely (100%) saponified and converted to pinanediol and sodium carbonate (Scheme 4). Base hydrolysis of the α-pinane carbonate *via* reactions with hydroxide ions to form carbonic acid salt and α-pinanediol is shown in Scheme 4. This reaction proceeds similarly to typical ester saponification. It should be noted that such reactions can also be performed using different hydroxide ion solutions, such as aqueous hydroxide solutions,^46^ or hydroxide solutions prepared in a mixture of water and soluble organic solvents, including dimethyl sulphoxide (DMSO),^47^ ethanol,^48,49^ dichloromethane^50^ and other suitable solvents.

**Scheme 5:**
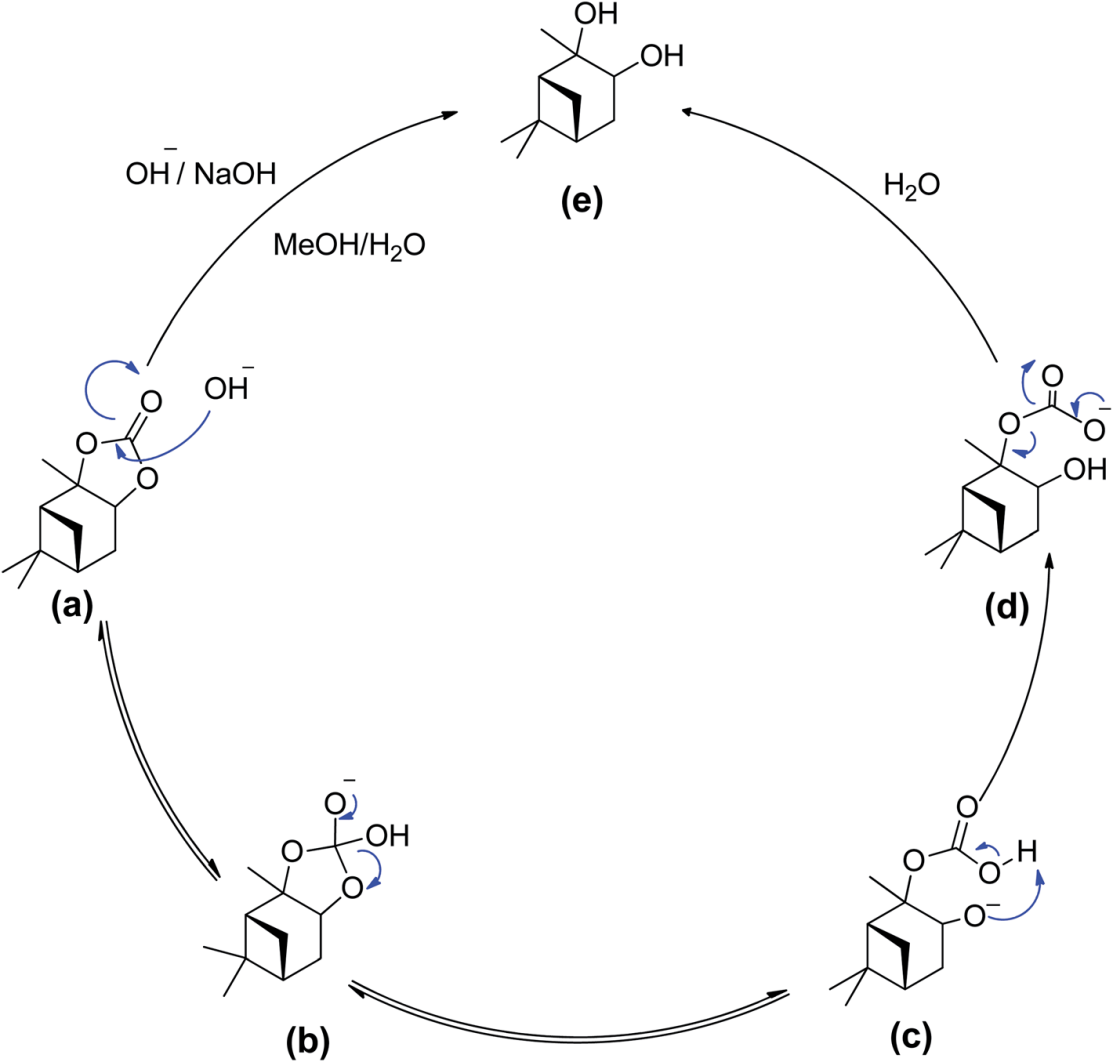
Proposed reaction mechanism – a bimolecular collision of solvated hydroxide ion with the alkyl esters carbonyl carbon.

The use of ethanol/water systems (*i.e.*, water and soluble organic solvents) allows for a complete dissolution of the NaOH and the α-pinane carbonate, which eliminates the mass transfer limitations that can occur due to poor immiscibility of carbonate esters and water. To ensure complete saponification of the α-pinane carbonate, the NaOH solution was used in a large excess of the stoichiometric amounts.^39^ A proposed reaction mechanism for base hydrolysis of the α-pinane carbonate is shown in Scheme 5, with a rate-determining step involving the bimolecular collision of the pinane carbonate and hydroxide ion to form a tetrahedral intermediate, which decomposes to give the products, as previously reported for saponification of alkyl esters.^51^

In this reaction mixture, a molecule of water is required to stabilise the intermediates and the same can also be provided by other protic solvents such as simple alcohols, like base hydrolysis of alkyl esters.^50,51^ During the reaction as shown in the Scheme 5, the hydroxyl group acted as a nucleophile and reacted with α-pinane carbonate (a) to yield intermediate (b) which decomposes and the carbonyl group reforms to form 2-carboxylate-3-pinacol (d) *via* intermediate (c) in the basic ethanol/water solution that further rearranges to furnish final product (e). As one side of α-pinane carbonate is more crowded thus it is most likely that the hydroxyl group as a nucleophile attacks on the opposite side on the most electropositive position however work is in progress to determine the exact mechanism of the reaction. Overall, the α-pinane carbonate hydrolysis was rapid, leading to the quantitative recovery of the α-pinanediol within 30 min. Previously, studies have shown that the reactivity of carboxylic acids decreases with the length of alkyl substituent due to a combination of polar and steric influences of the alpha substituent on the carboxylic group.^52–54^ The carbonic acid moiety on the α-pinane carbonate has no alkyl substituent, hence, its base hydrolysis is rapid. It is envisaged that poly(α-pinane carbonate) would follow a similar reaction pattern at slower reaction rates due to higher molecular weight.

## 4. Conclusions

This study is the first case of synthesis of α-pinane carbonate from α-pinanediol, a derivative of the most abundant naturally occurring monoterpene, α-pinene. Previous attempts to produce pinane carbonate by reactions of α-pinene epoxide with CO_2_ have been unsuccessful due to the lack of reactivity of the epoxide in ring-opening polymerisation because of steric hindrance. Here, α-pinane carbonate was synthesised by the reactions of α-pinanediol with DMC in the presence of a TBD guanidine catalyst. The α-pinane carbonate is a white crystalline solid with 86 °C melting point. The maximum α-pinane carbonate yield achieved was 81.1 ± 2.8%, at 98.7% purity, after recrystallization. The α-pinane carbonate produced was characterised using FTIR, NMR (^1^H and ^13^C), GC and GCMS and QTOF mass spectrometer. Reactivity of the α-pinane carbonate towards base hydrolysis with NaOH in ethanol/water was investigated to develop a feasible route for possible recovery and recycling of the α-pinanediol starting material. It was shown that α-pinane carbonate could be completely saponified within 30 min to the α-pinanediol and sodium carbonate. The α-pinane carbonate can likely be polymerised to obtain poly(α-pinane carbonate), which would be a bio-based thermoplastic, like poly(limonene carbonates) in an industrial scale process. The used poly(α-pinane carbonate) could be hydrolysed to recycle the α-pinanediol starting feedstock in the same way as the α-pinane carbonate monomer.

## Conflicts of interest

There are no conflicts to declare.

## Supplementary Material

RA-012-D1RA07943C-s001
