# Supplementary material for: Synthesis of cyclic α-pinane carbonate – a potential monomer for bio-based polymers
Source: RSC Adv. 2022 Jun 13;12(27):17454–65. doi: 10.1039/d1ra07943c (PMC9192141; doi:10.1039/d1ra07943c)
Supplement: RA-012-D1RA07943C-s001 [file RA-012-D1RA07943C-s001.pdf]

Electronic Supplementary Material (ESI) for RSC Advances.
This journal is © The Royal Society of Chemistry 2022

# Electronic Supplementary Information

The DSC thermal analysis in **ESI** Fig. 1 shows some evidence of gradual loss in heat flow after the melting point, as the nitrogen purge gas removes developing sample vapours. Therefore, this method could not be used to measure the normal boiling points of the samples, which requires an equilibrium vapour formation at an atmospheric pressure.

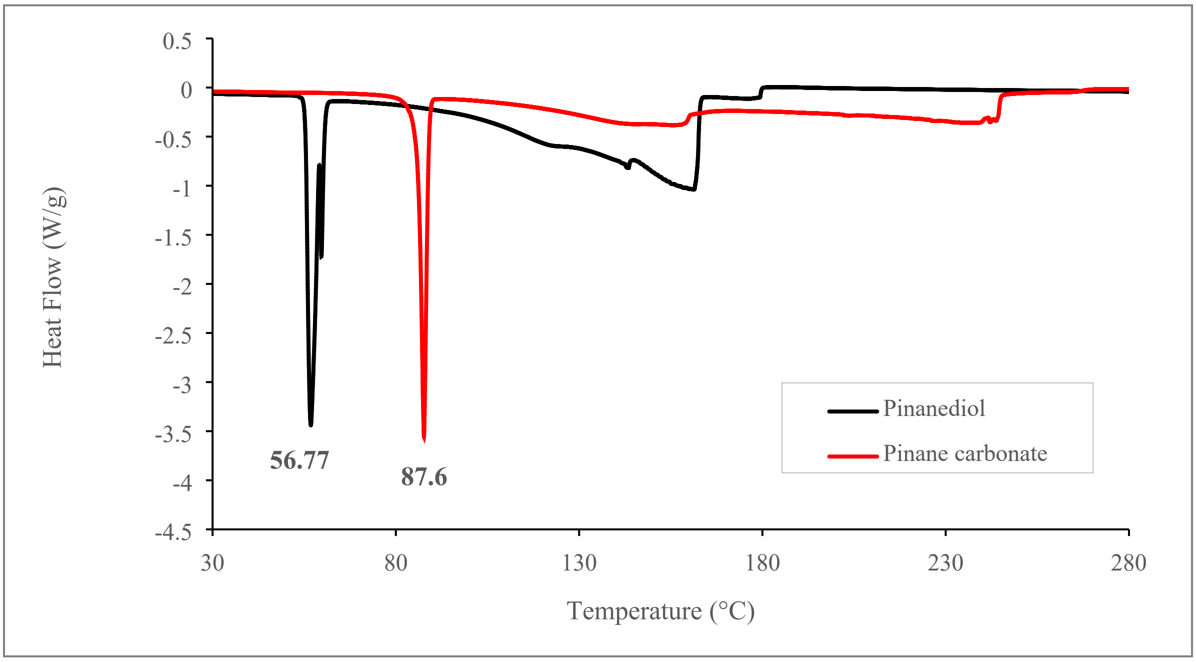


**ESI** Figure 1: DSC data for determinations of the melting points of the pinanediol and the pinane carbonate.

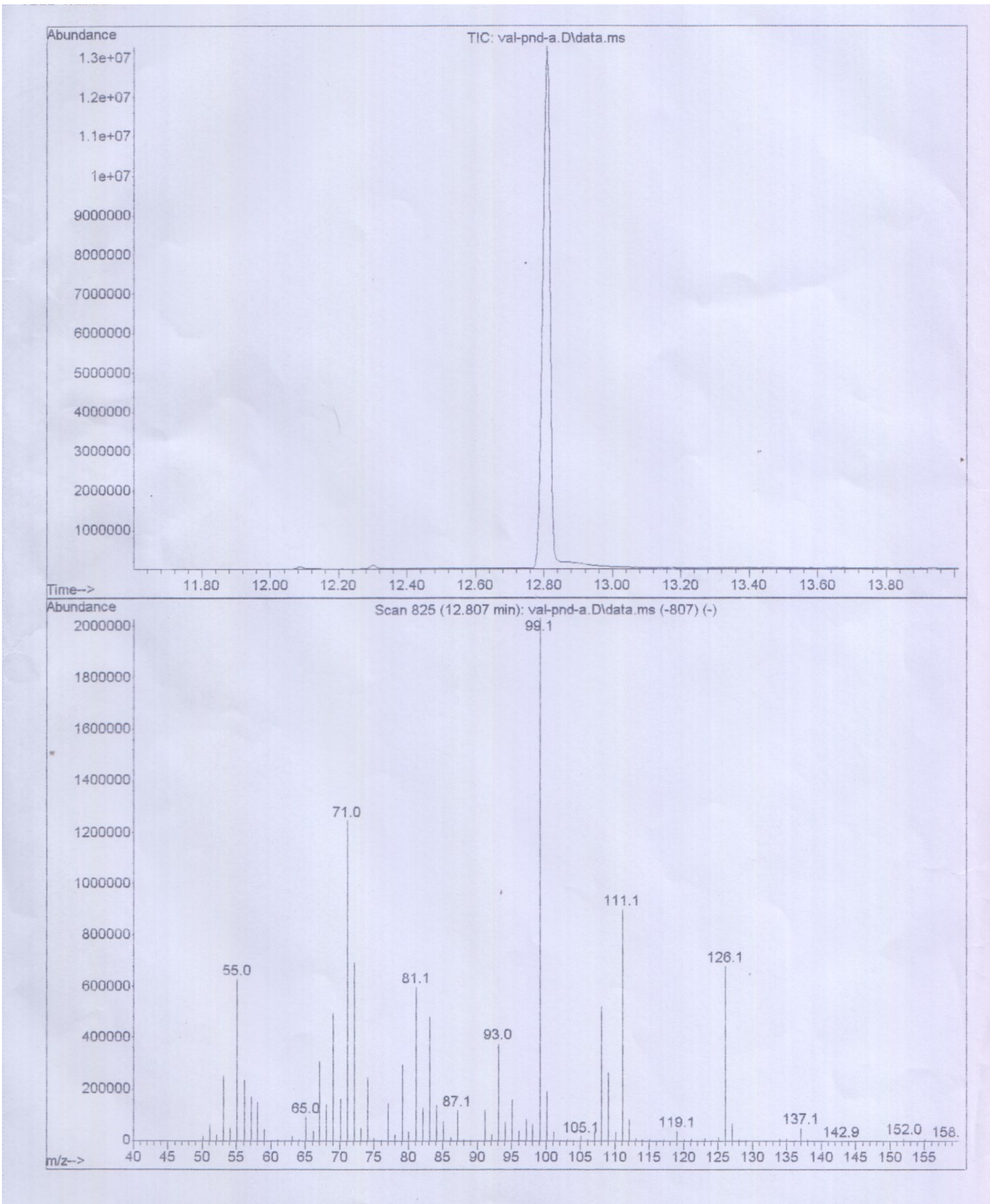


**ESI** Figure 2: GCMS data for the α-pinanediol acquired using a 7890B Agilent GC coupled to a 5977B mass selective detector (MSD) in full scan mode from 50-520amu and 70 eV.

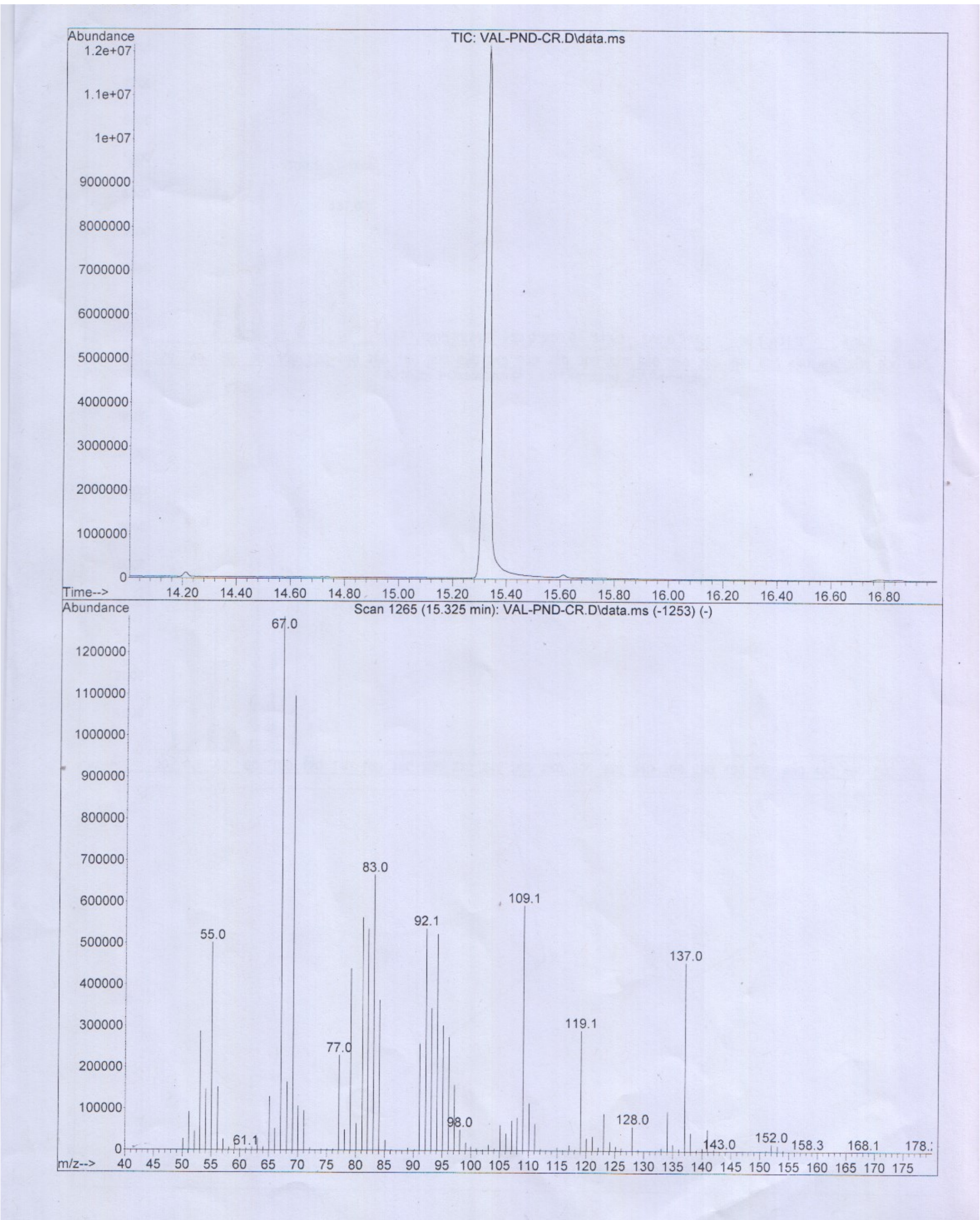


**ESI** Figure 3: GCMS data for the α-pinane carbonate acquired using 7890B Agilent GC coupled to a 5977B mass selective detector (MSD) in full scan mode from 50-520amu and 70 eV.

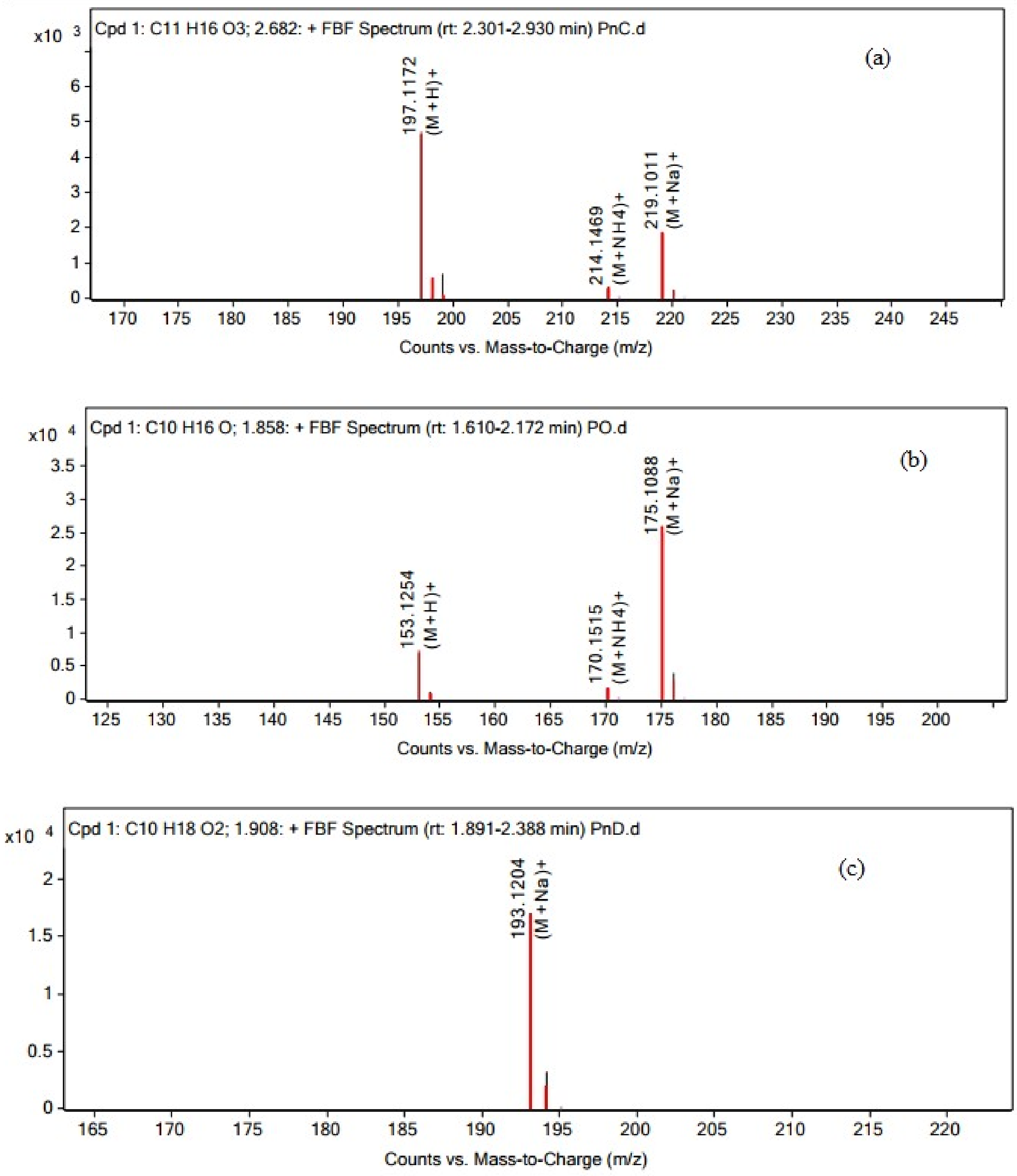


**ESI** Figure 4: QTOF MS data for the (a) α-pinane carbonate, (b) α-pinene epoxide and (c) α-pinanediol.

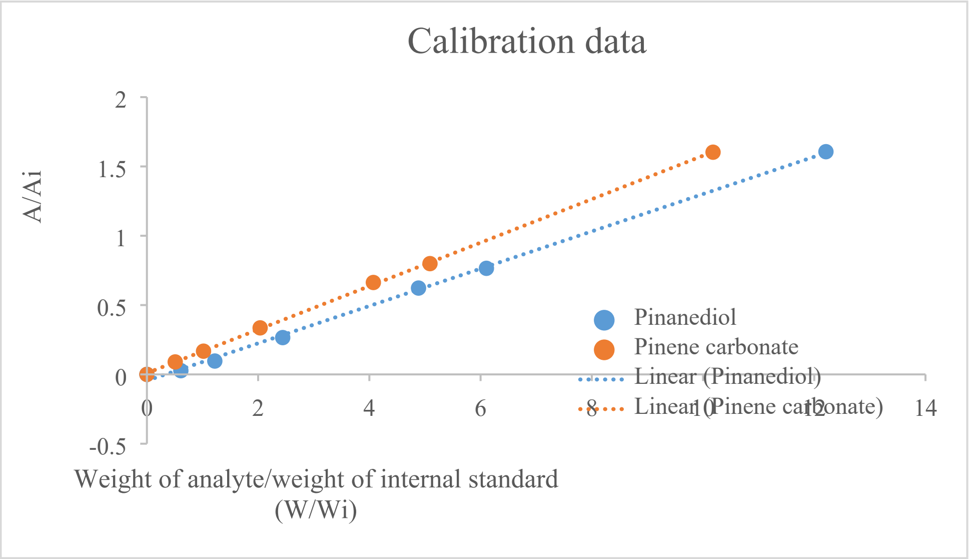


**ESI** Figure 5: Calibration data for the α-pinane carbonate and α-pinanediol.
